# Zinc Transporter SLC39A7/ZIP7 Promotes Intestinal Epithelial Self-Renewal by Resolving ER Stress

**DOI:** 10.1371/journal.pgen.1006349

**Published:** 2016-10-13

**Authors:** Wakana Ohashi, Shunsuke Kimura, Toshihiko Iwanaga, Yukihiro Furusawa, Tarou Irié, Hironori Izumi, Takashi Watanabe, Atsushi Hijikata, Takafumi Hara, Osamu Ohara, Haruhiko Koseki, Toshiro Sato, Sylvie Robine, Hisashi Mori, Yuichi Hattori, Hiroshi Watarai, Kenji Mishima, Hiroshi Ohno, Koji Hase, Toshiyuki Fukada

**Affiliations:** 1 RIKEN Center for Integrative Medical Sciences, Yokohama, Kanagawa, Japan; 2 Department of Molecular and Medical Pharmacology, Graduate School of Medicine and Pharmaceutical Sciences, University of Toyama, Sugitani, Toyama, Japan; 3 Laboratory of Histology and Cytology, Graduate School of Medicine, Hokkaido University, Sapporo, Hokkaido, Japan; 4 Division of Biochemistry, Faculty of Pharmacy, Keio University, Minato-ku, Tokyo, Japan; 5 Division of Mucosal Barriology, International Research and Development Center for Mucosal Vaccines, The Institute of Medical Science, The University of Tokyo, Minato-ku, Tokyo, Japan; 6 Division of Pathology, Department of Oral Diagnostic Sciences, School of Dentistry, Showa University, Shinagawa-ku, Tokyo, Japan; 7 Department of Molecular Neuroscience, Graduate School of Medicine and Pharmaceutical Sciences, University of Toyama, Sugitani, Toyama, Japan; 8 Laboratory for Integrative Genomics, RIKEN Center for Integrative Medical Sciences, Yokohama, Kanagawa, Japan; 9 Nagahama Institute of Bio-Science and Technology, Tamura, Nagahama, Shiga, Japan; 10 Molecular and Cellular Physiology, Faculty of Pharmaceutical Sciences, Tokushima Bunri University, Yamashiro, Tokushima, Japan; 11 Department of Technology Development, Kazusa DNA Research Institute, Kisarazu, Chiba, Japan; 12 Laboratory for Developmental Genetics, RIKEN Center for Integrative Medical Sciences, Yokohama, Kanagawa, Japan; 13 Department of Gastroenterology, School of Medicine, Keio University, Shinjuku-ku, Tokyo, Japan; 14 Equipe de Morphogenese et Signalisation cellulaires UMR 144 CNRS/Institut Curie, Paris, France; 15 Division of Stem Cell Cellomics, Center for Stem Cell Biology and Regenerative Medicine, The Institute of Medical Science, The University of Tokyo, Minato-ku, Tokyo, Japan; 16 Laboratory for Intestinal Ecosystem, RIKEN Center for Integrative Medical Sciences, Yokohama, Kanagawa, Japan; Faculty of Health Sciences, Ben-Gurion University of the Negev, ISRAEL

## Abstract

Zinc transporters play a critical role in spatiotemporal regulation of zinc homeostasis. Although disruption of zinc homeostasis has been implicated in disorders such as intestinal inflammation and aberrant epithelial morphology, it is largely unknown which zinc transporters are responsible for the intestinal epithelial homeostasis. Here, we show that Zrt-Irt-like protein (ZIP) transporter ZIP7, which is highly expressed in the intestinal crypt, is essential for intestinal epithelial proliferation. Mice lacking *Zip7* in intestinal epithelium triggered endoplasmic reticulum (ER) stress in proliferative progenitor cells, leading to significant cell death of progenitor cells. *Zip7* deficiency led to the loss of *Olfm4*^*+*^ intestinal stem cells and the degeneration of post-mitotic Paneth cells, indicating a fundamental requirement for *Zip7* in homeostatic intestinal regeneration. Taken together, these findings provide evidence for the importance of ZIP7 in maintenance of intestinal epithelial homeostasis through the regulation of ER function in proliferative progenitor cells and maintenance of intestinal stem cells. Therapeutic targeting of ZIP7 could lead to effective treatment of gastrointestinal disorders.

## Author Summary

Intestinal epithelium undergoes continuous self-renewal to maintain intestinal homeostasis. Given that dysregulation of zinc flux causes intestinal disorders, appropriate spatiotemporal regulation of zinc in the intracellular compartments should be a prerequisite for the intestinal epithelial self-renewal process. Zinc transporters such as Zrt-Irt-like proteins (ZIPs) are essential to fine-tune intracellular zinc flux. However, the link between specific zinc transporter(s) and intestinal epithelial self-renewal remains to be elucidated. Here, we found that ZIP7 is highly expressed in the intestinal crypts. The finding motivated us to further analyze the role of ZIP7 in intestinal homeostasis. ZIP7 deficiency greatly enhanced ER stress response in proliferative progenitor cells, which induced apoptotic cell death. This abnormality disrupted epithelial proliferation and intestinal stemness. Based on these observations, we reason that ZIP7-dependent zinc transport facilitates the vigorous epithelial proliferation in the intestine by ameliorating ER stress.

## Introduction

The intestinal epithelium, which renews every 3–5 days, is one of the most rapidly self-renewing tissues in adult mammals [1]. Homeostasis of the intestinal epithelium requires a fine balance between cell proliferation, migration, differentiation, and death [1]. Intestinal epithelial cells (IECs) are generated by intestinal stem cells, which are slender columnar cells that are interspersed with Paneth cells at the base of the intestinal crypt. Intestinal stem cells are characterized by expression of specific markers such as *Lgr5*, *Olfm4*, and *Ascl2* [2–5]. They divide to form transit-amplifying (TA) cells, which are localized to the lower part of the crypt [2]. TA cells divide continuously, and the daughter cells differentiate into absorptive enterocytes and secretory cell lineages: goblet cells, enteroendocrine cells, and Paneth cells.

Secretory epithelial cells have been shown to be sensitive to endoplasmic reticulum (ER) stress due to excessive protein synthesis of mucin and antimicrobial products [6,7]. Several mouse models with defects in protein folding or the unfolded protein response (UPR) exhibit enhanced ER stress in secretory cell lineages, which causes intestinal inflammation [6,8]. Furthermore, genetic mutation of the UPR transcription factor *Xbp1*, which is required for maintaining secretory cell lineages, is associated with a risk for developing inflammatory bowel disease [6]. UPR may also be important in regulating the differentiation of intestinal epithelial stem cells since it is induced to resolve ER stress during the transition from stem to TA cell. Moreover, inducing excessive ER stress affects stemness [9]. These observations indicate that UPR signals from the ER are critical for maintaining epithelial homeostasis in the intestine.

The ER acts as an intracellular store for biological mediators, including zinc, which is released into the cytoplasm in response to extracellular stimuli, such as IgE receptor cross-linking in mast cells [10,11]. Zinc deficiency causes ER stress in yeast and mammalian cells [12], indicating that a subcellular abundance of zinc may be crucial for ensuring cellular homeostasis when ER stress is physiologically induced, particularly in the intestinal epithelium [9]. Specific zinc transporters are responsible for the spatiotemporal regulation of intracellular zinc storage. Zinc transporters are classified into two major families, SLC39A/ZIP and SLC30A/ZnT. The ZIP family of proteins facilitate zinc influx to the cytoplasm from extracellular spaces as well as intracellular compartments, including the ER, whereas the ZnT family of proteins transport zinc in the opposite direction [13]. Recently, we and others have demonstrated the involvement of SLC39A/ZIP family members in a variety of cellular functions, including cell proliferation, differentiation, survival, and migration [14–21].

Among ZIP family members, ZIP7 is an intracellular zinc transporter that localizes to the ER and is a potential target of Wnt/β-catenin [22,23]. It is notably upregulated in breast cancer cells as well as a gastric tumor model [22]. Considering that Wnt/β-catenin signaling governs vigorous TA cell proliferation as well as the maintenance of intestinal stem cells, we hypothesized that a ZIP7-mediated zinc signal might contribute to epithelial homeostasis. Here, we demonstrate that the ER-localized zinc transporter, ZIP7, plays a key role in vigorous IEC proliferation, and in maintaining intestinal stemness, by alleviating ER stress in TA cells. Our data indicate that the fine-tuning of intracellular Zn homeostasis by ZIP7 is indispensable for epithelial proliferation and maintenance of stemness in the intestine.

## Results

### ZIP7 expression is enriched in TA and Paneth cells at intestinal crypts

**Fig 1:**
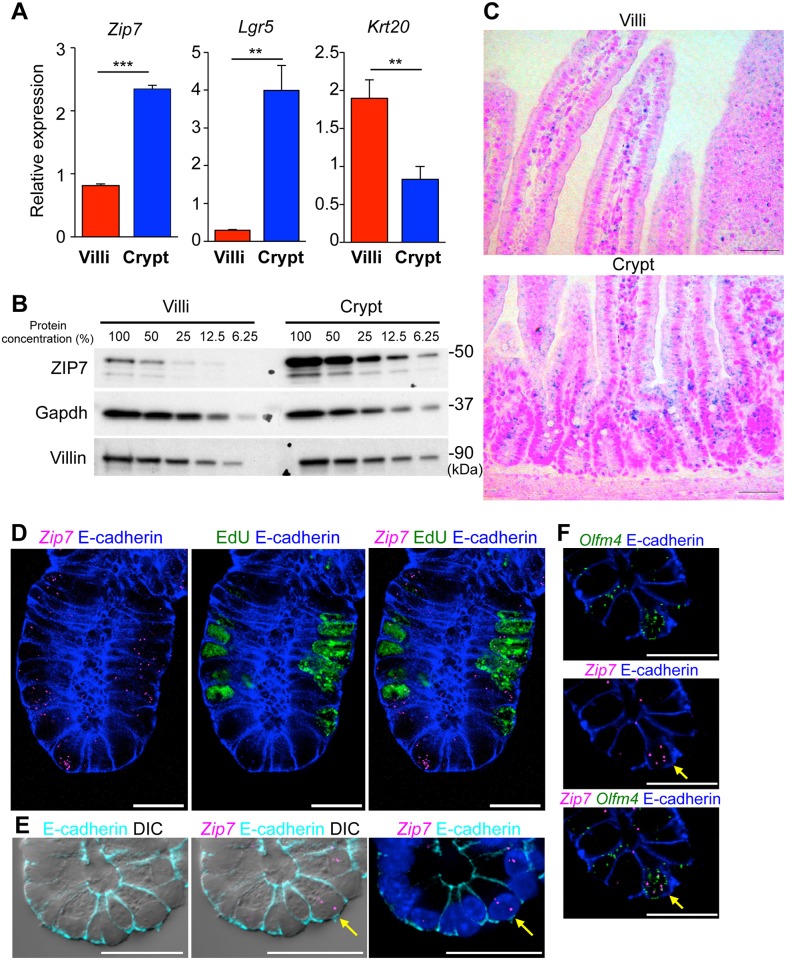
ZIP7 distribution in the mouse small intestine. (A) Quantitative PCR analysis of the relative expression of *Zip7*, *Lgr5*, and *Krt20* in villi and crypt. (B) ZIP7 protein levels in villi and crypt were analyzed by western blot of serial dilutions of villi and crypt protein lysates. (C) *In situ* hybridization of *Zip7* in small intestine tissues. Scale bar: 50 μm. (D) Simultaneous analysis of *in situ* hybridization of *Zip7* (magenta), EdU staining (green) and immunohistochemical E-cadherin staining (blue). Left: merged images of *Zip7* and E-cadherin. Center: merged images of EdU and E-cadherin. Right: merged images of *Zip7*, EdU and E-cadherin. Mice were injected with EdU 2 h before sacrifice. Cell borders were visualized by E-cadherin staining. Scale bar: 20 μm. (E) Simultaneous analysis of *in situ* hybridization of *Zip7* (magenta) and immunohistochemical E-cadherin staining (cyan). Left panel: merged images of E-cadherin and DIC. Center panel: merged images of *Zip7* (magenta), E-cadherin (Cyan) and DIC. Right panel: merged images of DIC, *Zip7*, E-cadherin and Hoechst33342. Arrows indicate Paneth cells. (F) Simultaneous analysis of *in situ* hybridization of *Zip7* (magenta), *Olfm4* (green) and immunohistochemical E-cadherin staining (blue). Top panel: merged images of *Olfm4* (green) and E-cadherin. Middle panel: merged images of *Zip7* (magenta) and E-cadherin. Bottom panel: merged images of *Zip7* (magenta), *Olfm4* (green) and E-cadherin (blue). Arrows indicate *Olfm4*-positive stem cells. Scale bar: 20 μm.

To characterize ZIP7 distribution in the mouse intestine, we first analyzed the villous and crypt epithelium from the small intestine by quantitative PCR. Consistent with previous reports, *Lgr5* [2] and *Krt20* [24] were highly expressed in the crypts and the villi, respectively. *Zip7* expression was enriched in the crypts (Fig 1A); this was confirmed by immunoblotting for ZIP7 proteins (Fig 1B).

*In situ* hybridization analysis demonstrated that *Zip7* was distributed in the middle and lower crypt regions in a pattern similar to that of TA cells (Fig 1C and S1 Fig). Multi-color FISH analysis demonstrated that *Zip7* was positive for the EdU-incorporated TA cells at the lower part of crypt (Fig 1D). *Zip7* expression was also detected by the cells with typical Paneth-cell morphology represented by intracellular granules (Fig 1E, arrows) and *Olfm4-*positive cells at the bottom of crypts (Fig 1F, arrows). Collectively, *Zip7* was highly expressed in premature proliferative cells, stem cells, and post-mitotic Paneth cells, but its expression was lower in the villous epithelium.

### *Zip7* deficiency severely impairs the epithelial integrity and regeneration of the intestine

**Fig 2:**
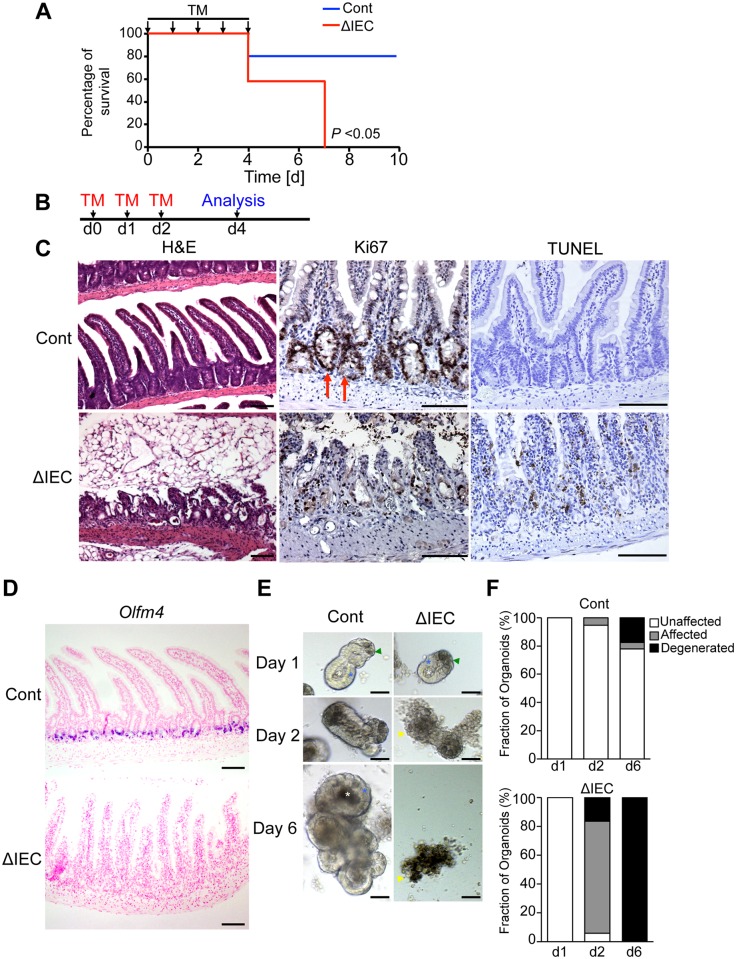
ZIP7 is required for homeostasis of the intestinal epithelium. (A) Survival curves of *Zip7*^Cont^ (Cont: *Zip7*^Cont^, *Zip7*^flox/+^*/Vil-CreERT2*, blue line, n = 5) and *Zip7*^ΔIEC^ (ΔIEC: *Zip7*^ΔIEC^, *Zip7*^flox/flox^*/Vil-CreERT2*, red line, n = 7) mice. Tamoxifen (TM) was administered orally for five consecutive days (arrows) to induce *Zip7* deletion. (B) TM protocol used in Fig 2C. (C) H&E (Left), Ki67 staining (center), and TUNEL staining (right) of *Zip7*^Cont^ and *Zip7*^ΔIEC^ intestines. Deleting *Zip7* led to substantial damage to the intestine (left panels), loss of proliferative progenitors (center panels), and apoptosis (right panels). Scale bar: 100 μm. (D) Identification of *Olfm4*^*+*^ stem cells by *in situ* hybridization of *Olfm4* in the small intestines from *Zip7*^Cont^ and *Zip7*^ΔIEC^ mice. In the *Zip7*^ΔIEC^ intestine, *Olfm4*^+^ stem cells were lost after *Zip7* deletion. (E) Morphology of small intestinal organoids from *Zip7*^Cont^ or *Zip7*^ΔIEC^ mice. *Zip7*^Cont^ crypts (Cont) proliferated and formed budding structures during the first 6 days of culture. *Zip7*^ΔIEC^ crypts (ΔIEC) treated with 4-OHT progressively degenerated. Representative images from three independent experiments. Scale bar: 50 μm. (F) Quantification of the fate of organoids in E. The status of organoids was traced for 6 days. Organoids were scored as unaffected (white bars), affected (gray bars), or degenerated (black bars). (Cont Day 1: n = 83; Cont Day 2: n = 73; Cont Day 6: n = 63; ΔIEC Day 1: n = 96; ΔIEC Day 2: n = 120; ΔIEC Day 6: n = 42).

To investigate the role of ZIP7 in epithelial homeostasis, we generated a mouse line with floxed alleles of *Zip7* (*Zip7*^flox/flox^; S2A Fig). *Zip7*^flox/flox^ mice were crossed with *Villin* (*Vil*)-*CreERT2* Tg mice [25] to generate *Zip7*^ΔIEC^ mice, in which the *Zip7* gene can be deleted in IECs by administering tamoxifen (*Zip7*^ΔIEC^). Adult *Zip7*^ΔIEC^ mice and control littermates carrying a heterozygous floxed allele (*Zip7*^flox/+^*/Vil-CreERT2;* referred to as *Zip7*^Cont^) were given tamoxifen orally for five consecutive days. About half of the *Zip7*^ΔIEC^ mice died by day 4, and all were dead by day 7 (Fig 2A). We subsequently treated the mice with tamoxifen for three consecutive days and performed histological examination at day 4. In this protocol, more than 80% of *Zip7*^ΔIEC^ mice remained alive. Deleting *Zip7* impaired epithelial integrity and led to the loss of the proliferating compartment (Fig 2B and 2C). TdT-mediated nick end labeling (TUNEL) assays revealed increased numbers of apoptotic cells in *Zip7*^ΔIEC^ intestine (Fig 2C). Crypt base columnar cells (CBC cells) marked by *Lgr5* or *Olfm4* are regarded as mitotically active intestinal stem cells and produce all epithelial cell lineages, including the proliferative progeny. Because of the loss of Ki67-positive cells in the crypts, we speculated that ZIP7 may affect the CBC population. In support of this notion, *Olfm4*^+^ stem cells at the bottom of crypts disappeared in *Zip7*^ΔIEC^ mice within 3 days of tamoxifen treatment (Fig 2D). These observations demonstrate that ZIP7 is essential for intestinal epithelial proliferation and maintenance of intestinal stem cells.

To further corroborate the requirement of *Zip7* for intestinal epithelial proliferation and maintenance of intestinal stem cells, independent of the niche, we established a crypt-derived organoid from *Zip7*^Cont^ and *Zip7*^ΔIEC^ mice, and examined the influence of *Zip7* deficiency on organoid growth in an *in vitro* culture system. Using a conventional method [26], small-intestinal crypts were isolated from *Zip7*^ΔIEC^ and *Zip7*^Cont^ mice, embedded in Matrigel, and cultured with 4-hydroxytamoxifen (4-OHT) for 48 h (S3 Fig). Crypts derived from control mice underwent crypt fission on day 2, and eventually generated epithelial organoids with multiple budding crypts on day 6 (Fig 2E) [26]. In contrast, 4-OHT-treated crypts from *Zip7*^ΔIEC^ mice failed to undergo crypt fission and died within 6 days (Fig 2E and 2F). Following the 4-OHT treatment, *Zip7*^ΔIEC^ organoids initially appeared morphologically intact, characterized by epithelial (Day 1 in Fig 2E, blue asterisk) and luminal (Day 1 in Fig 2E, white asterisk) compartmentalization and the presence of Paneth cells (Day 1 in Fig 2E green arrowhead). Then, *Zip7*^ΔIEC^ organoids generated cellular debris, which covered large areas of the surface of the organoids (Affected organoid, Day 2 in Fig 2E, yellow arrowhead), and finally, *Zip7*^ΔIEC^ organoids degenerated (Day 6 in Fig 2E, yellow arrowhead). Ex vivo *Zip7*-deficient crypt cells are not able to self-renew or survive (Fig 2F). These data indicate that ZIP7 is a prerequisite for the homeostatic regeneration of intestinal crypts.

### *Lgr5*^*+*^ cell-intrinsic ZIP7 secures stemness after radiation injury

To further analyze the importance of *Lgr5*^*+*^ cell-intrinsic ZIP7 on intestinal stemness, we generated *Lgr5*^*EGFP*^*-IRES*-*CreERT2*/*Zip7*^flox/flox^ (Zip7^ΔLgr5^) mice. *Lgr5*^+^ stem cell-specific deletion of ZIP7 affected neither epithelial homeostasis (S4A Fig) nor crypt-derived epithelial organoid formation (S4B Fig). Thus, these results suggest that *Lgr5*^+^ stem cell-intrinsic ZIP7 plays a redundant role in the homeostatic self-renewal under physiological conditions. Previous studies have demonstrated that *Lgr5*^+^ stem cells are dispensable for homeostatic self-renewal of intestinal epithelium [27,28]; however, this cell population is required for regeneration after damage such as irradiation [27]. We therefore asked the importance of ZIP7 in *Lgr5*^+^ stem cells in the regenerative process. *Zip7*^ΔLgr5^ and *Zip7*^cont^ mice received 7.5-Gy whole body irradiation after tamoxifen treatment (S4C Fig). While half of *Zip7*^cont^ mice survived over 50 days after the irradiation, all of *Zip7*^ΔLgr5^ mice died within 4 days (S4D Fig). Based on these observations, ZIP7 in *Lgr5*^+^ stem cells is required for intestinal regeneration after radiation damage.

### *Zip7* deficiency causes degeneration of Paneth cells

**Fig 3:**
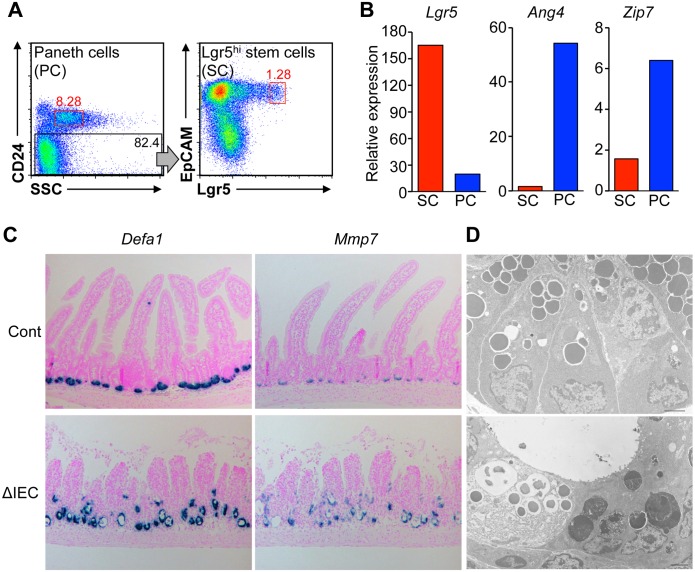
Loss of *Zip7* leads to degeneration of Paneth cells. (A) Dissected Paneth cells and *Lgr5*^+^ intestinal stem cells were sorted by flow cytometry into CD24^hi^SSC^hi^ (Paneth cells) and CD24^-^EGFP^+^EpCAM^hi^ (stem cells) populations. (B) Quantitative PCR analysis of *Lgr5*, *Ang4* (Paneth cell marker), and *Zip7* genes in sorted stem cell (SC) and Paneth cell (PC) populations. (C) Identification of Paneth cells by *Defa1* (blue) and *Mmp7* (blue) *in situ* hybridization analysis in small intestine from *Zip7*^Cont^ and *Zip7*^ΔIEC^ mice. (D) Transmission electron microscopic analysis of the small intestine from *Zip7*^Cont^ and *Zip7*^ΔIEC^ mice, 3 days after tamoxifen treatment. Scale bar: 2 μm.

The bottom of small intestinal crypts contains post-mitotic Paneth cells juxtaposed to intestinal stem cells. Because *Zip7* expression is higher in Paneth cells than in stem cells (Fig 3A and 3B), we analyzed the influence of ZIP7 deficiency in Paneth cells. *In situ* hybridization analysis showed that Paneth cell markers, *Defa1* and *Mmp7*, were detected in *Zip7*^ΔIEC^ mice after tamoxifen treatment (Fig 3C). However, ultrastructural analysis revealed that *Zip7*-deficient Paneth cells had an abnormal morphology, characterized by destructive granules and a collapse of ER structure, and a part of Paneth cells underwent apoptosis (Fig 3D). In 100 crypts chosen at random from three *Zip7*^ΔIEC^ mice, more than 50% crypts displayed the complete loss of normal granule-containing Paneth cells and replacement with broken and/or dying Paneth cells (Fig 3D). This suggests that *Zip7* is indispensable for the maintenance of Paneth cells.

Paneth cells support the stem-cell niche by providing factors that maintain stemness, such as Wnt3, EGF and Notch ligands [29] and serve as essential niche cells in *in vitro* organoid models [30]. Crypt cells cannot form organoids in the absence of Paneth cells. It is therefore postulated that Paneth cell degeneration due to ZIP7 deficiency may affect self-renewal of organoids. To examine this postulation, the organoids are supplemented with Wnt3a, a Paneth cell-derived factor to maintain stem cells, which enable Paneth cell-null crypts to grow into organoids [30](S5A Fig). In *Zip7*-sufficient control crypts, exogenous Wnt3a caused the organoids to form enlarged, round cysts due to overgrowth (S5B Fig). However, exogenous Wnt3a did not support the growth of *Zip7*-deficient crypts, and failed to rescue crypts from cell death (S5B Fig). Therefore, we reasoned that the loss of self-renewal capacity is unlikely to be due to Paneth cell degeneration under physiological conditions in *Zip7*^ΔIEC^ mice.

### *Zip7* deficiency results in massive apoptosis of TA cells due to increased ER stress

**Fig 4:**
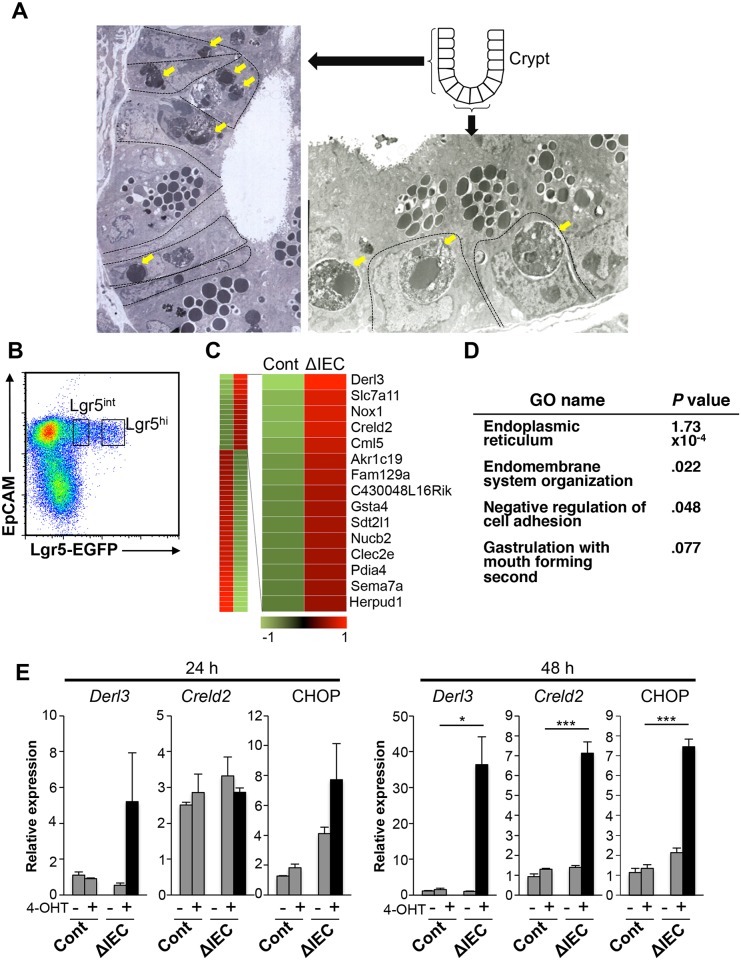
*Zip7* deletion activates the ER stress response and apoptosis in crypt cells. (A) Transmission electron microscopic analysis of the crypt of *Zip7*^ΔIEC^ small intestine 3 days after tamoxifen injection. Arrows indicate apoptotic cell bodies. (B) Flow cytometry of dissected stem and TA cells, sorted by Lgr5 expression into EpCAM^hi^Lgr5-EGFP^hi^ (stem cell) and EpCAM^hi^Lgr5-EGFP^int^ (TA cell) populations. (C) Heat map of 2.0-fold change of gene expression in sorted Lgr5^int^ TA cells from *Zip7*^Cont^ and *Zip7*^ΔIEC^ mice, 2 days after tamoxifen treatment. *Zip7* deletion induced ER stress-related gene expression in TA cells. Columns represent cell types and rows represent differentially expressed genes. (D) Gene ontology analysis of genes upregulated in TA cells from *Zip7*^ΔIEC^ small intestines. (E) Quantitative PCR analysis of *Derl3*, *Creld2* and CHOP in *Zip7*^Cont^ and *Zip7*^ΔIEC^ organoids 24 and 48 h after 4-OHT treatment. *P < 0.05 and *** *P* < 0.005 (Student’s *t*-test). Data are presented as mean ± SEM.

TA cells localize above the stem cell niche and vigorously proliferate before differentiation [2]. Transmission electron microscopy showed apoptotic bodies in the proliferative crypt compartment in the *Zip7*^ΔIEC^ small intestine 3 days after tamoxifen injection (Fig 4A, left panel), indicating that *Zip7* deficiency induced apoptosis in TA cells. Notably, apoptotic bodies were present inside CBC cells that were morphologically recognized as stem cells by a slender columnar shape interspersed with Paneth cells (Fig 4A, right panel). These observations raised the possibility that CBC cells may engulf neighboring dead cells as reported previously [31], although further study is necessary to confirm this possibility.

To clarify the intracellular events by which *Zip7* deficiency causes apoptosis of TA cells, we purified cell populations, based on EGFP expression intensity, from control [*Zip7*^Cont^*Lgr5*^EGFP^*-IRES-CreERT2*] and *Zip7*^ΔIEC^*Lgr5*^EGFP^*-IRES-CreERT2* mice 2 days after tamoxifen treatment (Fig 4B). The stem cell markers *Lgr5*, *Ascl2*, and *Olfm4* were exclusively expressed in the Lgr5^hi^ stem cell population and were downregulated in the Lgr5^int^ TA cell population (S6A Fig). Comparative gene expression profiling of intestinal TA cells showed that many UPR-related genes were more highly expressed in the *Zip7*-deficient TA cell population (Fig 4C and S6B Fig). Severe ER stress conditions activate apoptosis signaling through induction of pro-apoptotic factors such as *Trib3* and *Bcl2l15*. We also detected upregulation of these genes in the *Zip7*-deficient TA cells (S6B Fig). These results suggest that TA cell apoptosis can be attributed to severe ER stress as a result of *Zip7* deficiency. Gene ontology-based functional enrichment analysis also revealed that a group of ER function-related genes was upregulated in the *Zip7*-deficient TA cell population (Fig 4D). In agreement with these observations, *Zip7* ablation led to upregulation of the ER stress-related genes *Derl3* and *Creld2*, as well as *Ddit3* (encoding CHOP), which is responsible for an ER stress-dependent apoptotic pathway in cultured organoids (Fig 4E). Activation of ATF6 and the PERK pathway induces expression of transcription factor XBP1 (X-box-binding protein 1) [32]. Increased expression of *Xbp1* was observed in *Zip7*^ΔIEC^ organoids in response to exposure to 4-OHT (S7A Fig). Notably, *Mt1* whose expression depends upon intracellular zinc concentration was downregulated in *Zip7*^ΔIEC^ organoids (S7B Fig), suggesting that the intracellular zinc may be decreased in the organoid. We therefore examined whether exogenous supplementation of zinc could ameliorate ER stress caused by *Zip7* deficiency; however, the zinc supplementation failed to suppress the induction of ER-stress-related genes (S8A Fig). Collectively, these results indicate that ablating ZIP7 augments ER stress and eventually induces apoptosis in TA cells. Therefore, ZIP7 ensures ER function in TA cells, which is essential for maintaining the vigorous proliferative properties of TA cells. Importantly, the same effect was seen in *Zip7*-deficient *Lgr5*^+^ stem cells (S6C Fig), suggesting that ZIP7 is also a key regulator of ER function in intestinal stem cells.

### ZIP7 regulates UPR signaling

**Fig 5:**
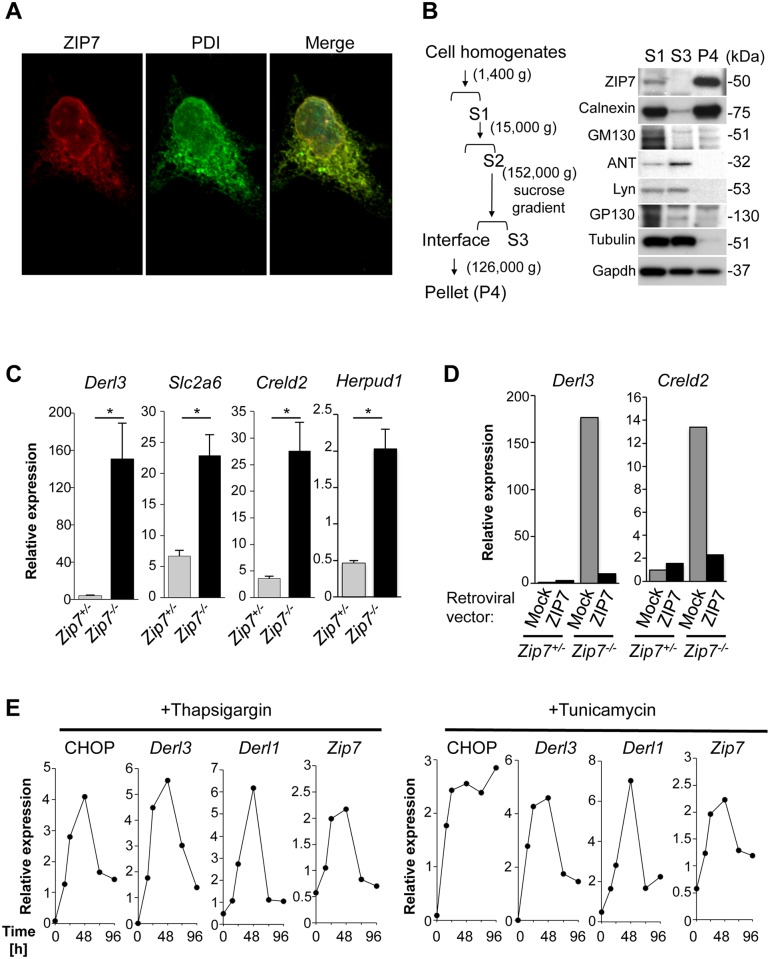
ZIP7 is necessary for resolving ER stress responses. (A) ZIP7 subcellular localization. ZIP7 colocalizes with PDI in 293T cells. V5-tagged ZIP7 and PDI were stained with V5 (red) and PDI (green) antibodies. PDI was used as an ER marker. (B) (Left) Flow diagram for subcellular fractionation. NIH-3T3 cells were homogenized and centrifuged to obtain the supernatant (S1), which was centrifuged and subjected to discontinuous sucrose gradient fractionation. The supernatant (S3) and interface fraction were collected. The interface fraction was further centrifuged to obtain the ER-enriched pellet (P4). (Right) Immunoblots of subcellular fractions. ZIP7 was enriched in a fraction containing ER but not Golgi or mitochondria. Calnexin (ER), GM130 (Golgi), adenine nucleotide translocator (ANT, mitochondria), Lyn and GP130 (Plasma membrane), Tubulin (cytoskeleton), and Gapdh (cytosol) were used as markers for various organelles. (C) Quantitative PCR analysis of ER stress-related genes, *Derl3*, *Slc2a6*, *Creld2 and Herpud1* in control (*Zip7*^*+/-*^, *Zip7*^flox/+^/*Rosa26-CreERT2*) or *Zip7*^-/-^ (*Zip7*^flox/flox^*/Rosa26-CreERT2*) MEF cells treated with 1 μM 4-OHT for 48 h. ER stress genes were upregulated in *Zip7*^*-/-*^ cells. (D) Overexpression of ZIP7 in MEF cells rescued ER stress gene (*Derl3*, *Creld2*) expression induced by *Zip7* deletion. *Zip7*^*+/-*^ or *Zip7*^*-/-*^ MEF cells were infected with mock or ZIP7-expressing retroviruses, treated with 1 μM 4-OHT for 48 h, and analyzed by quantitative PCR. (E) Thapsigargin or tunicamycin upregulated the transcription of *Zip7* and ER stress marker genes (CHOP, *Derl3*, and *Derl1*) in MEF cells. Cells were treated with 0.25 μM thapsigargin or 0.5 μg/mL tunicamycin for the indicated periods. Data are presented as mean ± SEM from at least three independent experiments.

We next explored the molecular mechanism by which ZIP7 ameliorates ER stress. ImmunofluoZIP7 was mainly co-localized with the ER-resident protein, PDI (Protein disulfide-isomerase), at the perinuclear region (Fig 5A). Subcellular fractionation by sucrose gradient-based ultracentrifugation confirmed that ZIP7 predominantly fractionated with the ER (Fig 5B); this localization raised the possibility that ZIP7 was involved in maintenance of ER homeostasis. To examine this possibility, we prepared mouse embryonic fibroblasts (MEFs) from *Zip7*^flox/flox^*/Rosa26-CreERT2* mice (*Zip7*^*-/-*^ in Fig 5C and 5D), in which *Zip7* can be deleted with 4-OHT treatment (S2B and S2C Fig). Comparative gene expression profiling of MEF cells demonstrated that UPR-related genes, such as *Derl3*, *Slc2a6*, *Creld2*, *Herpud1 and Ddit3* (encoding CHOP), were upregulated in *Zip7*^*-/-*^ MEF cells (S9 Fig). Quantitative PCR analysis confirmed that the upregulation of the UPR-related genes (Fig 5C, S8B and S9 Figs). Exogenous zinc supplementation did not affect the upregulation of UPR in *Zip7*^-/-^ MEF cells (S8B Fig) as observed in *Zip7*^ΔIEC^ organoids (S8A Fig). On the other hand, exogenously expressed wild-type ZIP7 rescued UPR caused by the loss of ZIP7 (Fig 5D). Intriguingly, *Zip7* expression increased in response to tunicamycin- or thapsigargin-induced ER stress (Fig 5E), suggesting that ZIP7 supports homeostatic ER functions and protects against ER stress by regulating UPR-related genes.

## Discussion

**Fig 6:**
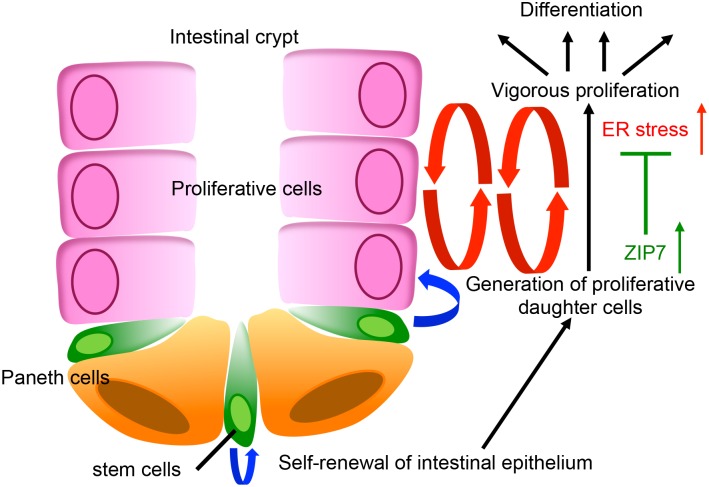
A schematic model of the ZIP7-dependent rapid epithelial proliferation in intestine. Intestinal stem cells located at the bottom of the crypt self-renew and generate proliferative cells. These cells proliferate vigorously, producing large numbers of differentiated cells. During this proliferation, ZIP7 is induced and resolves the upregulated ER stress, thereby ensuring proliferative responses in intestinal crypts.

Here, we demonstrate that the ER-localized zinc transporter, ZIP7, is indispensable for the vigorous proliferation of TA cells, and for maintaining the stemness of intestinal stem cells. Specifically, ZIP7 was abundantly expressed in the crypt epithelium, and deleting *Zip7* led to the loss of both TA and stem cells as well as to increased ER stress responses and UPR in crypts. The UPR, which is triggered by high bioenergetic demands, is important in helping TA and stem cells adapt to the demands of biosynthesis and in preventing ER stress-induced apoptosis. Therefore, our study demonstrates that ZIP7 controls crypt homeostasis and strongly contributes to ER function in TA cells (Fig 6).

We observed massive apoptotic cell death in the middle of crypts in *Zip7*^ΔIEC^ mice, where apoptotic bodies derived from TA cells had been incorporated into stem cells (Fig 4A). Studies show that irradiation- or chemotherapy-induced apoptotic cells are incorporated into neighboring stem cells [31,33], and cells undergoing apoptosis generate signals that induce apoptosis in neighboring cells, leading to cohort cell death [34,35]. Given these observations, deleting *Zip7* may cause massive apoptosis in crypt cells, particularly TA cells, with the apoptotic bodies being taken up by adjacent viable stem cells. The apoptosis of neighboring cells and accumulation of apoptotic bodies appear to be harmful for stem cells, as evidenced by their upregulation of ER stress molecules. These findings suggest that stem cells may be lost since a large number of neighboring cells are undergoing apoptosis due to *Zip7* deletion.

Deleting *Zip7* induces the expression of UPR genes (Fig 4C and 4E and S6B Fig), indicating that the failure of *Zip7*-deficient TA cells to resolve ER stress leads to the upregulation of UPR genes and to apoptosis. UPR signaling is associated with IEC proliferation and differentiation. For example, activation of UPR signaling promotes the differentiation of stem cells into TA cells [9]. XBP1, a key component of the ER stress response, is required for secretory cell development and maintenance [6]. ER stress regulators and UPR signaling molecules are upregulated in TA cells compared to stem cells [9]. Notably, TA cells strongly expressed ZIP7, which was upregulated by ER stress (Fig 5E). UPR-dependent upregulation of ZIP7 in TA cells might resolve ER stress, thereby securing the highly proliferative status of TA cells. These results suggest that zinc concentration in the ER is tightly regulated by ZIP7, and in TA cells, this regulation is a prerequisite to resolving ER stress.

*Lgr5*^*+*^ stem cells are well documented to show resistance to irradiation, because stem cells can repair DNA damage more efficiently than radio-sensitive TA cells and the other epithelial cell types of the small intestine [24,36]. Indeed, repopulation after radiation damage begins with CBCs, including *Lgr5*^+^ stem cells [36]. Depletion of *Lgr5*^*+*^ stem cells results in an impaired regenerative response after irradiation [27]. Thus, *Lgr5*^*+*^ stem cells are a prerequisite of the regenerative response for providing TA cells after epithelial damage. Nevertheless, *Zip7* deficiency in *Lgr5*^+^ stem cells remarkably increased susceptibility to irradiation due to a failure in regenerative responses. Our results suggest that ZIP7 critically contributes to the *Lgr5*^+^ cell-dependent regenerative response following irradiation. On the contrary, *Zip7* deficiency did not affect stemness without irradiation, implying that *Zip7* is dispensable for maintenance of stem cells under the physiological conditions. It is noteworthy that *Lgr5*^+^ stem cells play a redundant role in homeostasis of the epithelium under physiological conditions, because *Bmi1*^*+*^ stem cells serve as an alternative stem cell pool[28]. We, therefore, do not formally exclude the possibility that *Bmi1*^*+*^ stem cells may compensate degeneration of *Zip7*-deficient *Lgr5*^+^ stem cells to maintain stemness.

We have also demonstrated that ZIP7 is essential for maintenance of Paneth cells that are susceptible to UPR. Ablation of *Xbp1* required for expansion and maintenance of the ER results in loss of Paneth cells [6]. We found that *Zip7* deletion induced ER stress in intestinal organoids, and *Zip7* was upregulated by ER stress induction. Paneth cells contain a large amount of zinc in their granules[37], and perturbation of zinc homeostasis elicits extrusion of Paneth cells[38]. Furthermore, upregulation of *Zip7* expression is associated with the development of Paneth cells [39]. Collectively, ZIP7-mediated regulation of zinc homeostasis seems to be responsible for maintenance of Paneth cells. Because Paneth cells serve as niche cells for intestinal stem cells by providing EGF, Wnt and Notch ligands[29], this cell population is required for the *in vitro* organoid culture system that lacks mesenchymal cells, the major producer of Wnt and Notch ligands in the intestine, as evidenced by the observation that the Paneth cell-null crypts fail to develop to organoids[30]. This abnormality can be rescued by exogenous Wnt3a supplementation[30]. We observed that Wnt3a supplementation did not improve the growth arrest in *Zip7*^ΔIEC^ organoids. This observation supports the notion that loss of intestinal stem cells in *Zip7*^ΔIEC^ organoid was unlikely attributed to degeneration of Paneth cells.

Although the precise mechanisms by which ZIP7 resolves ER stress in the intestinal epithelium are unclear, several key phenomena and predictions may help answer this question. For instance, ZIP7 is needed to maintain sufficient zinc concentration in order to meet the requirements of ER-resident proteins that use zinc as a cofactor, such as ER-localized calreticulin, which binds monoglucosylated carbohydrate on newly synthesized glycoproteins [40,41]. In addition, the flexible coordination geometry of zinc [42] and facile ligand exchange allow it to bind proteins stochastically to substitute for a different native metal ion [43]. Therefore, stochastic binding of zinc to ER-resident proteins could negatively affect protein conformation and function. For example, protein disulfide isomerase, which regulates protein folding by catalyzing disulfide bonds in the ER, is oligomerized in the presence of zinc and thus has reduced catalytic activity [44]. Excessive zinc accumulation in the ER may lead to ectopic binding to various ER proteins, thus impairing their structure and function. Misfolded proteins are transported across the ER membrane for cytosolic proteasome degradation in a process known as ER-associated degradation (ERAD) [45,46]. Key ERAD components include E3 ubiquitin ligases that are embedded in the ER membrane [47]. Most ERAD E3 ligases possess a zinc-coordinating RING domain to facilitate E2-dependent ubiquitylation [47]. The formation of a rigid, globular platform for protein-protein interactions in RING fingers requires zinc [47], implying that fine-tuning zinc concentration by ZIP7 seems to be essential for normal ER function. UPR activation induces the expression of ERAD components. Similarly, *Zip7* expression was elevated in cells treated with tunicamycin or thapsigargin to induce UPR (Fig 5E), suggesting that ZIP7 may be involved in an adaptive program to alleviate ER stress. ZIP7 is reported to be phosphorylated and activated by casein kinase 2 (CK2), an ER-localized serine/threonine protein kinase [48]. CK2 also plays a key role in the ER stress response [49]. Therefore, the CK2-ZIP7-zinc signaling axis may moderate the strength of UPR signaling.

In this study, we illustrate that ZIP7 is fundamental to the homeostatic and active proliferation of the intestinal epithelium and the maintenance of stem cells in the crypt. Our findings also indicate that ZIP7 is a potential therapeutic target for gastrointestinal tumors. Given that various cells proliferate vigorously under certain physiological and pathological conditions, our study represents an important step toward uncovering the mechanisms by which cells adapt their intracellular conditions to maintain a brisk proliferative response through fine-tuning ER function.

## Materials and Methods

### Ethics statement

All mice were housed and cared for according to guidelines approved by the RIKEN Yokohama institutional Animal Care and Experiments committee (K24-007), or by the Committee for Institutional Animal Care and Use at the University of Toyama (A2015MED48).

### Animal experiments

All animal experiments were performed with the approval of the institutional Animal Care and Use committee of RIKEN IMS, and the Graduate School and Pharmaceutical Sciences, University of Toyama. *Zip7*^flox/flox^ mice were generated as previously described [50]. Briefly, we created a targeting vector to eliminate the genomic region encompassing exons 5 and 6 by inserting a loxP sequence and a *Neo* cassette into the region between exons 4 and 5, and a loxP sequence between exons 6 and 7 of *Slc39a7*. After this vector was introduced into R1 ES cells, cloned homologous recombinants were selected with antibiotics, and the genotypes were verified. We developed chimeric mice with the targeted ES cell clones and crossed them with *E2a-Cre* mice to delete the *Neo* cassette (S2 Fig). The mice used in experiments were backcrossed with C57BL/6J mice. *Villin (Vil)-CreERT2* transgenic mice were described previously [25]. *Lgr5*^*EGFP*^*-IRES*-*CreERT2* transgenic mice were obtained from Jackson Laboratories. *Rosa26-CreERT2* transgenic mice were purchased from Artemis Pharmaceuticals. The transgenic *Vil-CreERT2* or *Rosa26-CreERT2* mice were crossed with *Zip7*^flox/flox^ mice. To sort cells from *Zip7*^Cont^ and *Zip7*^ΔIEC^ mice based on Lgr5 levels, *Lgr5*^*EGFP*^-*IRES*-*CreERT2* transgenic mice were crossed with *Zip7*^flox/+^/*Vil-CreERT2* mice or *Zip7*^flox/flox^/*Vil-CreERT2* transgenic mice. Male and female mice were selected randomly for all experiments. *In vivo*, the Cre enzyme was activated by orally administering 100 μL tamoxifen (50 mg/mL) dissolved in corn oil/ethanol (9:1) for the indicated number of consecutive days. Genotyping was done by PCR using KOD-plus or KOD-FX NEO polymerases (Toyobo). The following primers were used for genotyping: 5’-CTTCATGCTTTACTGCCTCCGTTCC-3’ and 5’-ATAAATCCGCCTGCAGTGAA-3’.

### Antibodies

We purchased antibodies against Ki67 (Clone: MM1), Villin (Santa Cruz, sc-7672), Gapdh (Clone: 6C5), Calnexin (Clone: 37), GM130 (Clone: 35), Adenine nucleotide translocase (Santa Cruz, sc-9299), Lyn (Santa Cruz, sc-15), GP130 (Santa Cruz, sc-9045), Tubulin (Clone: B-5-1-2), EpCAM-PE (Clone: 9C4), CD24-APC (Clone: ML5), CD45-PE-Cy5 (Clone: 30-F11), and PDI (Clone, 1D3). We generated the antibody against mouse ZIP7 by immunizing a rabbit with a peptide corresponding to the GNTGPRDGPVKPQSPEE sequence of mouse ZIP7, which was affinity-purified by using the antigen peptide.

### Crypt isolation and three-dimensional culture

Mouse small intestines were isolated, opened longitudinally, and washed with cold PBS. The tissue was chopped into pieces approximately 5 mm in length and washed again with cold PBS. Tissue fragments were incubated in 1 mM dithiothreitol with Hank’s balanced salt solution (HBSS; Life Technologies) for 15 min on ice. After removing the dithiothreitol solution, the tissue fragments were washed with cold PBS and then with 2 mM EDTA with HBSS for 5 min, after which the samples were incubated in 2 mM EDTA with HBSS for 30 min on ice. Samples were then shaken vigorously, after which the supernatant was discarded. The sediment was washed into 5 mM EDTA with HBSS for 5 min on ice, incubated in 5 mM EDTA with HBSS for 30 min on ice, and shaken vigorously to yield free crypts. This fraction was passed through 100-μm and 70-μm cell strainers and then centrifuged at 150 *g* for 3 min. The isolated crypts were plated on Matrigel with culture medium (Advanced DMEM/F12, Invitrogen) containing the growth factors: EGF (50 ng/mL, Peprotech), R-spondin1 (500 ng/mL, R&D Systems), and noggin (100 ng/mL, Peprotech). The growth medium was changed every other day. In some experiments, Wnt3a-conditioned medium was added to the culture medium. For *in vitro* Cre activation, organoids were cultured with 1 μM 4-OHT for 48 h, after which the culture medium was replaced with fresh medium without 4-OHT. Three or more independent experiments were performed.

For quantification, the morphology of organoids was classified as follows: 1) Unaffected organoid with clearly distinguishable epithelial and luminal area under bright field; 2) Affected organoid that has cellular debris on a large part of the organoid surface and that is difficult to identify the luminal area inside the organoid; 3) Degenerated organoid that mostly consists of cellular debris and has no epithelial/luminal compartments.

### Immunohistochemistry and *in situ* hybridization

Intestinal samples were fixed and embedded in paraffin using standard protocols. Sections were deparaffinized in xylol and dehydrated in ethanol. Antigens were retrieved with citrate or EDTA buffer for 2 min at 105°C in an autoclave, and endogenous peroxidase was blocked. Primary antibodies were incubated overnight at 4°C, and secondary, horseradish peroxidase-conjugated antibodies were detected using a 3,3’-Diaminobenzidine Peroxidase Substrate Kit (Vector Laboratories). *In situ* hybridization for *Zip7*, *Olfm4*, and *Defa1* was performed by Genostaff (Tokyo, Japan) methods or by one of the methods described next.

### Fluorescent *in situ* hybridization (FISH) and immunohistochemistry

For labeling proliferating cells, mice were administered with EdU (5 mg/kg of body weight) by intraperitoneal injection. After 2 hours, the mice were sacrificed and the intestine was removed. Intestinal epithelial monolayer was isolated by modifying the method described by Kimura et al [51]. Briefly, the intestine were soaked in ice-cold Hank's balanced salt solution (Life Technologies) containing 30 mM EDTA and 5 mM dithiothreitol. After incubation with gentle shaking for 20 min on ice, epithelial monolayer was carefully separated from muscle layer by manipulation with a fine needle under stereomicroscopic monitoring. The isolated epithelium was fixed for 20 min in 4% paraformaldehyde in PBS at 4°C. The specimens were washed three times with 50 mM glycine in PBS, and then pretreated with 0.3% Triton X-100 in PBS for 10 min. Subsequently, EdU detection was carried out using the Click-iT EdU AlexaFluor488 imaging kit (Life Technologies) according to the manufacturer's protocol. Then, FISH was performed with the Quantigene View RNA ISH Cell Assay (Affymetrix, Santa Clara, CA) according to manufacturers' protocols. Specific oligonucleotide probe sets against *Slc39a7* (*Zip7*; VB6-14266) were purchased from Affymetrix, Inc. After FISH, samples were treated with an anti-E-cadherin (dilution 1:400 in PBS; AF748, R & D systems) goat polyclonal antibody overnight at room temperature and incubated with Cy3-conjugated anti-goat antibodies (1:1000; Life Technologies) for 2 h at room temperature. Specimens were mounted with SlowFade Gold Antifade Reagent (Life Technologies) and examined under a confocal laser microscope (FV1000, Olympus, Tokyo, Japan). Expression of Zip7 mRNA in the intestinal stem cells were analyzed by dual-color FISH with *Slc39a7* and *Olfm4* probes (VB1-11400) by similar procedure with the exception of EdU-labeling and detecting step.

### Electron microscopy

Mice were placed under deep anesthesia and perfused via the aorta with physiological saline followed by 2.5% glutaraldehyde in 0.1 M phosphate buffer at pH 7.4. The dissected tissues were immersed in fixative for another 3 h, briefly washed in phosphate buffer, post-fixed in 1% OsO_4_ for 90 min, dehydrated through a graded ethanol series, and embedded in Epon. Ultra-thin sections were prepared on an ultramicrotome, stained with uranyl acetate and lead citrate, and examined under an electron microscope (H-7100, Hitachi, Tokyo, Japan).

### cDNA cloning and plasmid construction

The murine *Zip7* gene was cloned by PCR using cDNA from the C57BL/6J mouse strain, sequenced on a 3130xI sequencer (ABI-PRISM, Applied Biosystems), and subcloned into the pcDNA6.2/V5-DEST expression vector (Invitrogen) to generate C-terminally V5-tagged mouse WT ZIP7. These constructs were transfected into 293T cells by Lipofectamine LTX reagent (Life technologies) according to manufacturer protocols.

### Cell culture and confocal microscopy

293T and MEF cells were maintained in DMEM or RPMI1640 medium containing 10% fetal bovine serum, penicillin, and streptomycin. The transfected cells were fixed, permeabilized, and stained with monoclonal anti-V5 and Dylight 488-conjugated anti-PDI antibodies. Anti-V5 was detected with polyclonal Alexa 546-conjugated goat anti-mouse IgG (Life technologies). Hoechst 33342 was used to visualize nuclei. Images were acquired by LSM780 confocal microscope (Zeiss)

### Quantitative RT-PCR

RNA was extracted with the RNAeasy Kit (Qiagen, UK) or Trizol (Life Technologies) or Sepazol (Nacalai Tesque) according to the manufacturers’ instructions. First-strand cDNA was synthesized by ReverTra Ace (Toyobo) according to the manufacturer’s instructions; mRNA levels were quantified by qPCR using SYBR Premix Ex Taq (Takara) or PowerUp SYBR Green Master Mix (ThermoFisher Scientific) and the ABI3100 or StepOnePlus system (Applied Biosystems) or MX3000p system (Stratagene), and were normalized to *Gapdh*. The sequences of the primers are provided in S1 Table.

### Immunoblotting

Isolated crypts, villi, or cells were lysed in radioimmunoprecipitation assay buffer. Lysates were clarified by centrifugation (30 min, 12,000 rpm, 4°C) and proteins were resolved by SDS-PAGE, electrotransferred onto polyvinylidene fluoride membranes, and immunoblotted. Horseradish peroxidase-bound secondary antibodies were detected with a chemiluminescence kit.

### Subcellular fractionation

Cells in HTE buffer (0.25 M sucrose, 10 mM Tris-HCl, 0.1 mM EDTA, pH 7.4) supplemented with protease inhibitor cocktail (Roche) were lysed by freeze-thawing. Nuclei and cellular debris were removed by centrifugation at 1,400 *g* for 10 min. Postmitochondrial supernatant obtained by centrifuging at 15,000 *g* for 10 min was layered on a sucrose step-gradient consisting of 1.3 M, 1.5 M, and 2.0 M sucrose in 10 mM Tris-HCl (pH 7.6) and banded by centrifugation at 152,000 *g* for 70 min. The ER fraction at the interface between the supernatant and the 1.3 M sucrose step was collected, diluted with HTE butter, and pelleted by centrifugation at 126,000 *g* for 45 min. The ER membranes were resuspended in HTE buffer.

### Retroviral infection

The Flag-tagged mouse ZIP7 plasmid was inserted into a pMX retroviral vector (a gift from T. Kitamura, The University of Tokyo, Tokyo, Japan). This construct was used to transfect the 293T-based Phoenix packaging cell line (a gift from G. Nolan, Stanford University, Stanford, CA). Lipofectamine 2000 (Invitrogen) was used to generate recombinant retroviruses. MEF cells were infected with the retrovirus in the presence of 10 μg/mL polybrene.

### Isolation of Paneth cells and Lgr5^+^ cells

Small intestinal crypts were isolated by EDTA chelation. Epithelial cells were dissociated using TrypLE express (Invitrogen) with 2,000 U/mL DNase I for 30 min at 37°C. Dissociated cells were passed through a 40-μm strainer, stained with anti-EpCAM-PE, anti-CD45-PE-Cy5, and anti-CD24-APC antibodies, and then analyzed by MoFlo. Dead cells were excluded by 7-AAD or SYTOX-AAD staining. Viable single cells were gated, and CD45^-^ CD24^+^ SSC^hi^, CD45^-^ CD24^-^ EpCAM^+^ Lgr5-EGFP^hi^, and CD45^-^ CD24^-^ EpCAM^+^ Lgr5-EGFP^int^ cells were sorted.

### Microarray analysis

RNA was isolated from the sorted Lgr5-EGFP^hi^ EpCAM^+^ CD45^-^ and Lgr5-EGFP^int^ EpCAM^+^ CD45^-^ cell fractions from the small intestines of *Zip7*^flox/+^/*Vil-CreERT2*/*Lgr5*^*EGFP*^*-IRES-CreERT2* or *Zip7*^flox/flox^/*Vil-CreERT2*/*Lgr5*^*EGFP*^*-IRES-CreERT2* mice. Microarray experiments were performed using Affymetrix Mouse Gene 1.0 ST Array GeneChips. Datasets were derived from three biological samples of each genotype. All microarray data have been deposited in the reference database of immune cells (RefDIC, http://refdic.rcai.riken.jp) under accession numbers: RSM13249, RSM13250, RSM13251, and RSM13252.

### Statistics

Differences in the means were examined by a 2-tailed unpaired Student’s *t*-test. Analyses were done on GraphPad Prism 6 (GraphPad Software). Results are presented as mean ± SEM or SD for the number of experiments indicated in each figure legend. Kaplan-Meier survival curves were compared using the log-rank test. *P* < 0.05 was considered statistically significant.

## Supporting Information

S1 Fig *In situ* hybridization analysis of *Zip7* in small intestine. *In situ* hybridization of *Zip7* in small intestine from C57BL/6J mice. Scale bar: 100 μm.
(TIF)

S2 Fig Generation of *Zip7*^flox/flox^ mice. (A) Targeting vectors with three LoxP insertions. In *Zip7*^flox^ mice, two LoxP sites were inserted into introns 4 and 6, respectively. *Zip7*^Δflox^ mice were obtained by crossing with Cre-transgenic mice. Gray triangles indicate LoxP sites; closed boxes indicate exons. (B) *Zip7* deletion. MEF cells derived from *Zip7*^*-/-*^ mice were treated with 1 μM 4-OHT for the indicated periods. NT, without 4-OHT. (C) Decreased ZIP7 protein levels in MEF cells from control (*Zip7*^flox/+^ *Rosa26-CreERT2*) or *Zip7*^*-/-*^ (*Zip7*^flox/flox^ *Rosa26-CreERT2*) mice after treatment with 1 μM 4-OHT. Tubulin and Gapdh were used as loading controls. TK, thymidine kinase.
(TIF)

S3 Fig Schematic diagram for intestinal organoid culture. Intestinal crypts isolated from *Zip7*^Cont^ and *Zip7*^ΔIEC^ mice were embedded in Matrigel and incubated with 1 μM 4-OHT.
(TIF)

S4 Fig *Zip7* in *Lgr5*^+^ stem cells is required for regeneration after irradiation. (A) Representative H&E staining of intestines from *Zip7*^Cont^ and *Zip7*^ΔLgr5^ mice. (B) Organoids established from *Zip7*^ΔLgr5^ mice with (lower panel) or without (upper panel) 4-OHT treatment for 48 h. (C) The tamoxifen (TM) induced-induced *Zip7* deletion and radiation ragimen used in D. (D) Survival curves of *Zip7*^Cont^ (n = 4) and *Zip7*^ΔLgr5^ (n = 4) mice treated with 7.5 Gy whole body irradiation. Tamoxifen (5 mg/kg i.p.) was treated 24 h before and after irradiation for 2 consecutive days.
(TIF)

S5 Fig ZIP7 governs organoid self-renewal independent of Paneth cells. (A) *In vitro Zip7*-deletion protocol to examine the effect of added Wnt3a on intestinal organoids from *Zip7*^Cont^ and *Zip7*^ΔIEC^ mice. (B) Crypts cultured in standard medium plus Wnt3a. Crypts from a control mouse showed rounded cysts. Exogenous Wnt3a did not support the growth of crypts from *Zip7*^ΔIEC^ mice. Scale bar: 50 μm.
(TIF)

S6 Fig RNA expression profiles of sorted Lgr5^hi^ and Lgr5^int^ cells. DNA microarray expression experiments were performed with sorted Lgr5^hi^ and Lgr5^int^ cells. (A) Heat map of stem cell-related genes in sorted Lgr5^hi^ and Lgr5^int^ cells from *Zip7*^Cont^ and Zip7^ΔIEC^ mice, showing a clear separation between the different Lgr5 populations. (B) Heat map of 1.8-fold change of gene epxression between *Zip7*^Cont^ and *Zip7*^ΔIEC^ Lgr5^int^ cells. (C) Heat map showing differentially expressed transcripts in sorted Lgr5^hi^ stem cells from *Zip7*^Cont^ and *Zip7*^ΔIEC^ mice 2 days after Cre activation. Columns represent different cell types, and rows represent differentially expressed genes.
(TIF)

S7 Fig *Zip7* deletion-induced gene expression changes. Quantitative PCR analysis of *Xbp1* (A) and *Mt1* (B) mRNA in cultured organoids from *Zip7*^Cont^ and *Zip7*^ΔIEC^ mice 48 h after 4-OHT treatment.
(TIF)

S8 Fig Effect of supplementation of Zn on *Zip7* deletion-induced upregulation of ER stress-related gene expression. (A) Quantitative PCR analysis of *Derl3*, CHOP, and *Creld2* in cultured organoids from *Zip7*^Cont^ and *Zip7*^ΔIEC^ mice 48 h after 4-OHT treatment with or without ZnSO_4_ (1 μM). (B) Quantitative PCR analysis of *Derl3* and CHOP *Zip7*^-/-^ MEF cells treated with 1μM of 4-OHT and ZnSO_4_ (1 μM).
(TIF)

S9 Fig RNA expression profiles of MEF cells from *Zip7*^+/-^ and *Zip7*^-/-^ mice. Heat map of 2.0-fold change of gene epxression between *Zip7*^+/-^ and *Zip7*^-/-^ MEF cells. Columns represent different cell types, and rows represent differentially expressed genes.
(TIF)

S1 Table Primer sequences of mouse genes used for quantitative real-time PCR. (PDF)
