# Supplementary figures and images for: Zinc Transporter SLC39A7/ZIP7 Promotes Intestinal Epithelial Self-Renewal by Resolving ER Stress
Source: PLoS Genet. 2016 Oct 13;12(10):e1006349. doi: 10.1371/journal.pgen.1006349 (PMC5065117; doi:10.1371/journal.pgen.1006349)

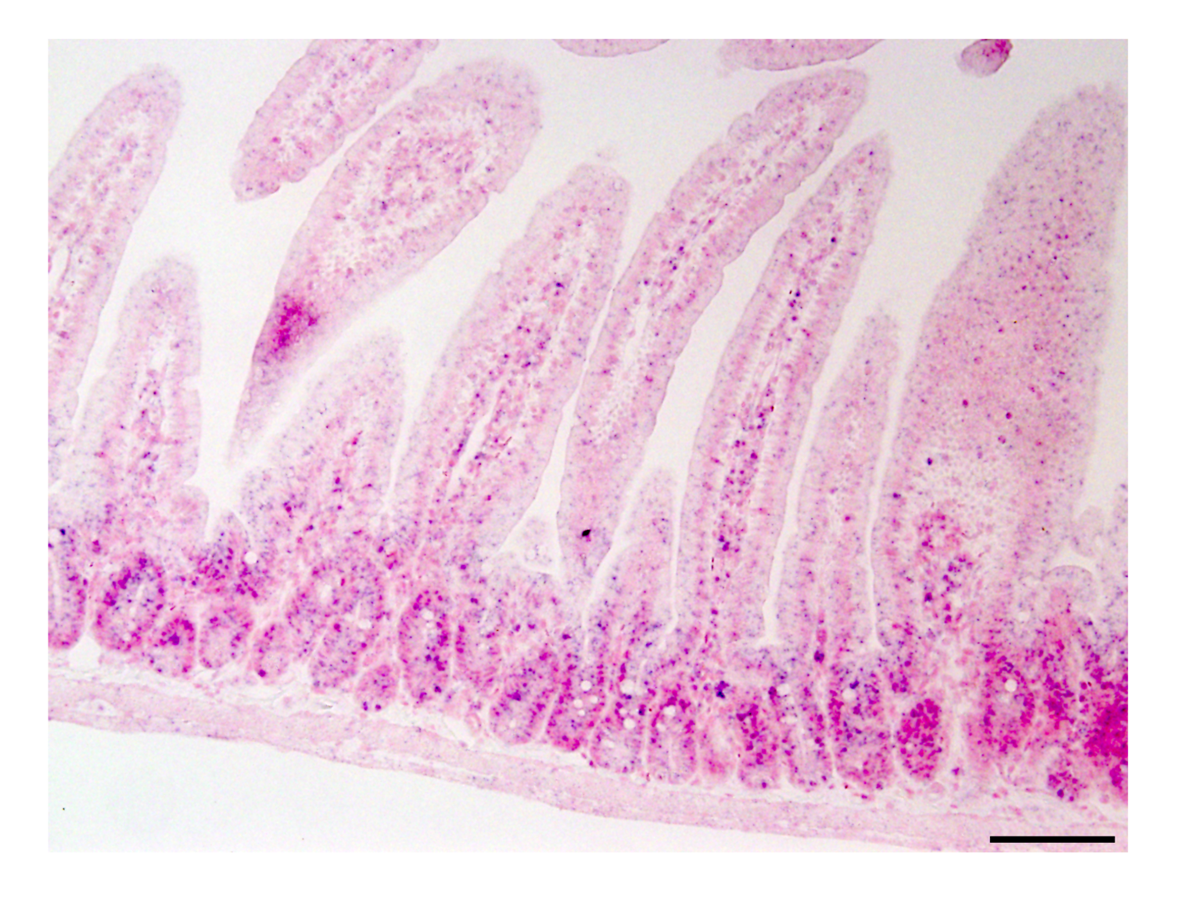

Supplement: S1 Fig — In situ hybridization of Zip7 in small intestine from C57BL/6J mice. Scale bar: 100 μm. (TIF) [file pgen.1006349.s001.tif]

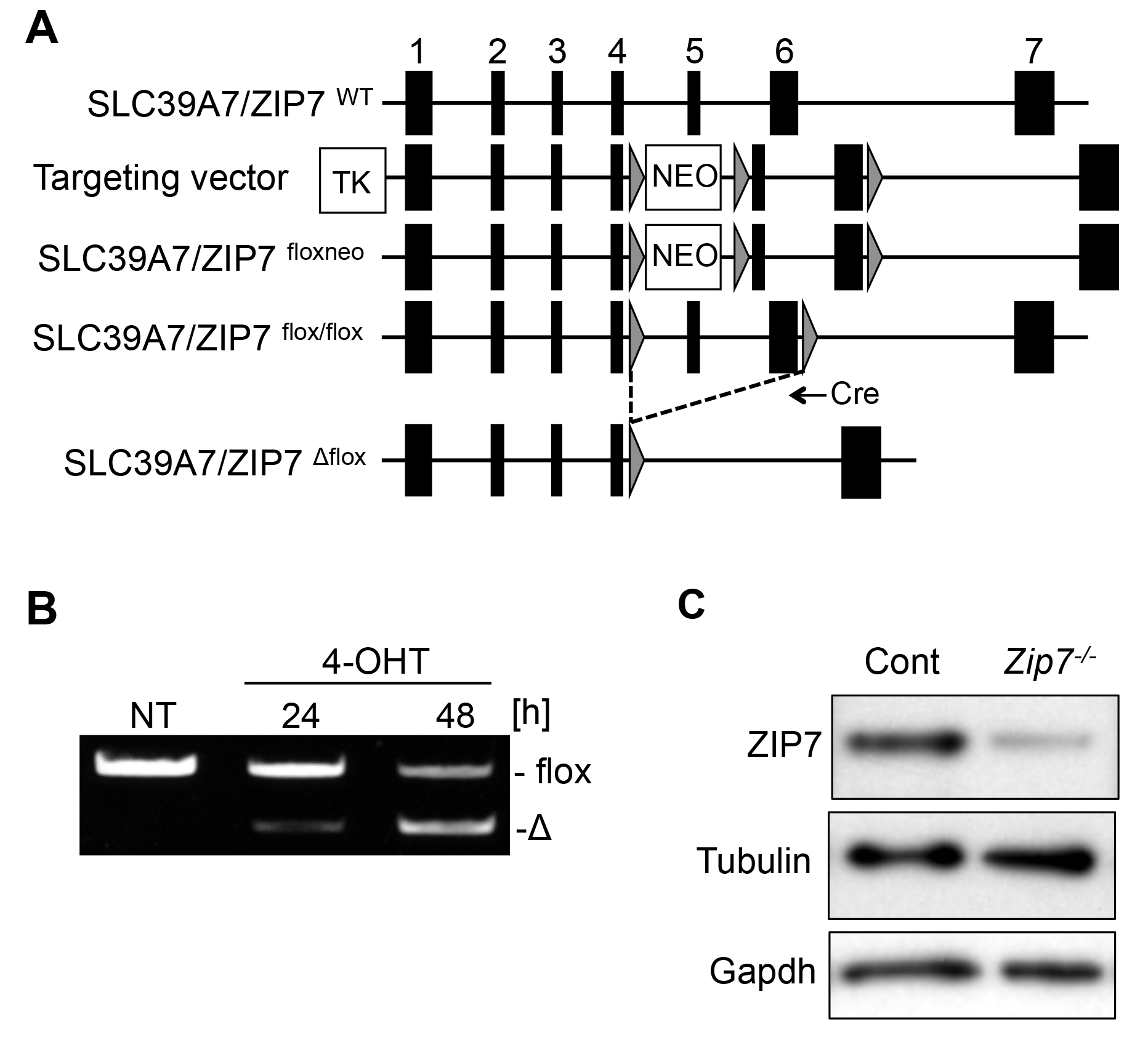

Supplement: S2 Fig — (A) Targeting vectors with three LoxP insertions. In Zip7flox mice, two LoxP sites were inserted into introns 4 and 6, respectively. Zip7Δflox mice were obtained by crossing with Cre-transgenic mice. Gray triangles indicate LoxP sites; closed boxes indicate exons. (B) Zip7 deletion. MEF cells derived from Zip7-/- mice were treated with 1 μM 4-OHT for the indicated periods. NT, without 4-OHT. (C) Decreased ZIP7 protein levels in MEF cells from control (Zip7flox/+ Rosa26-CreERT2) or Zip7-/- (Zip7flox/flox Rosa26-CreERT2) mice after treatment with 1 μM 4-OHT. Tubulin and Gapdh were used as loading controls. TK, thymidine kinase. (TIF) [file pgen.1006349.s002.tif]

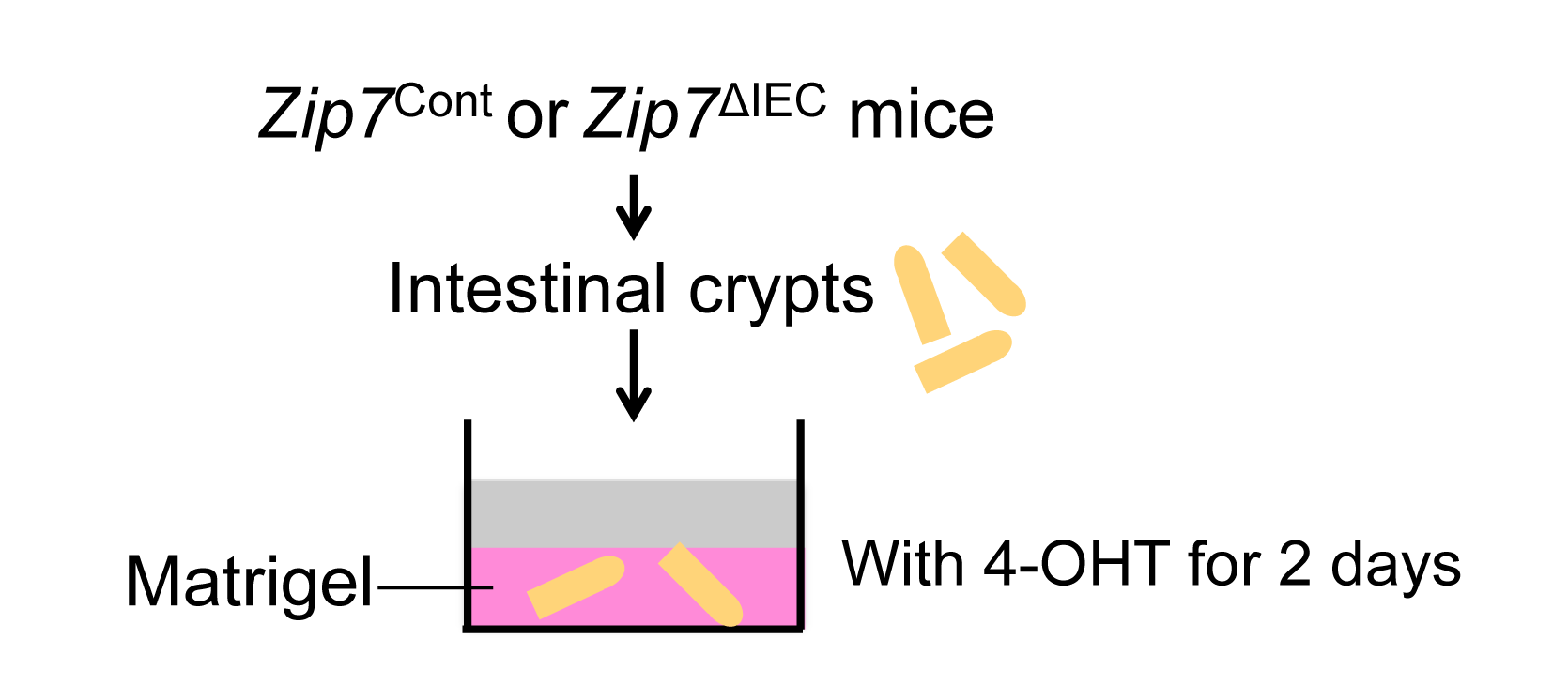

Supplement: S3 Fig — Intestinal crypts isolated from Zip7Cont and Zip7ΔIEC mice were embedded in Matrigel and incubated with 1 μM 4-OHT. (TIF) [file pgen.1006349.s003.tif]

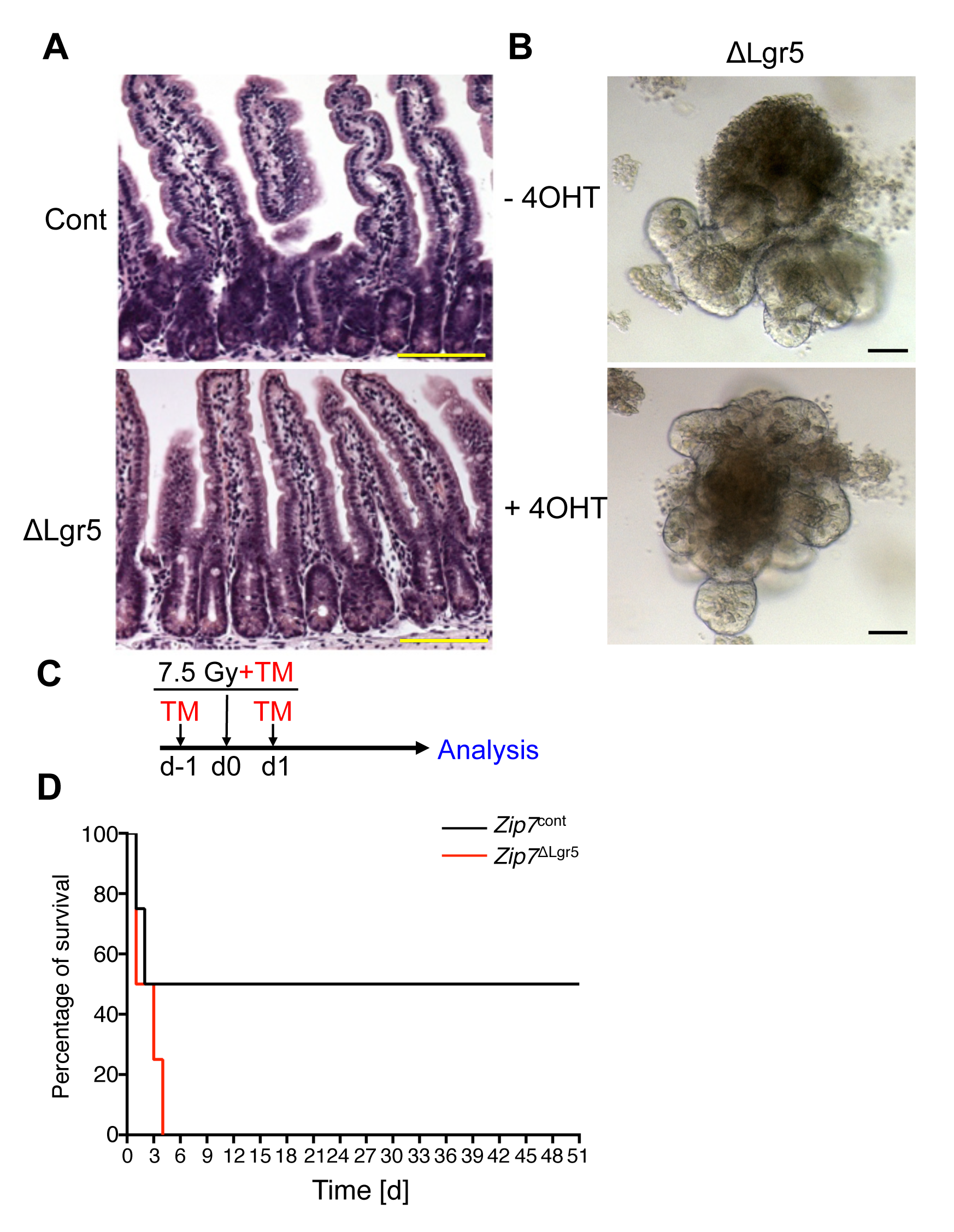

Supplement: S4 Fig — (A) Representative H&E staining of intestines from Zip7Cont and Zip7ΔLgr5 mice. (B) Organoids established from Zip7ΔLgr5 mice with (lower panel) or without (upper panel) 4-OHT treatment for 48 h. (C) The tamoxifen (TM) induced-induced Zip7 deletion and radiation ragimen used in D. (D) Survival curves of Zip7Cont (n = 4) and Zip7ΔLgr5 (n = 4) mice treated with 7.5 Gy whole body irradiation. Tamoxifen (5 mg/kg i.p.) was treated 24 h before and after irradiation for 2 consecutive days. (TIF) [file pgen.1006349.s004.tif]

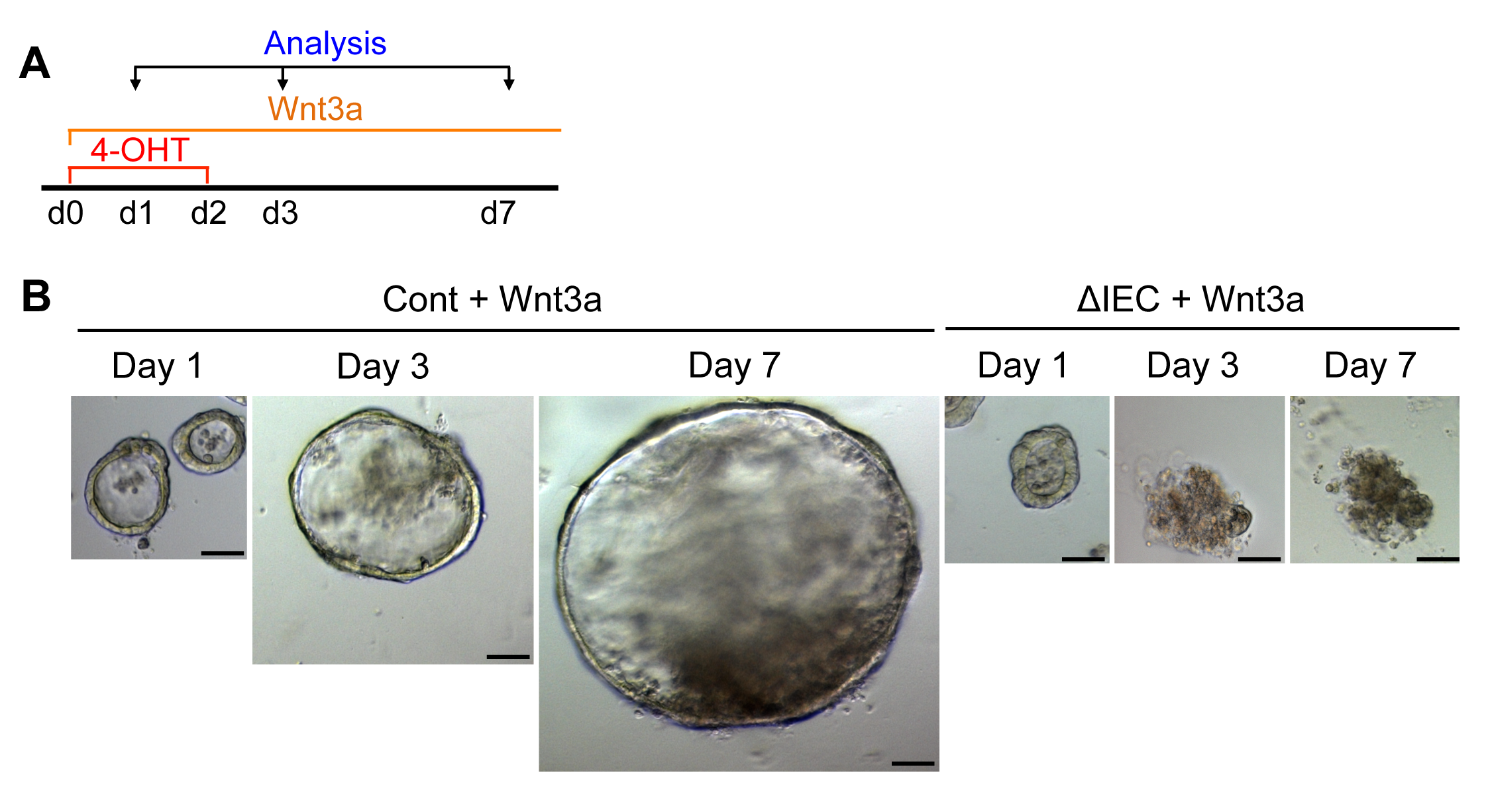

Supplement: S5 Fig — (A) In vitro Zip7-deletion protocol to examine the effect of added Wnt3a on intestinal organoids from Zip7Cont and Zip7ΔIEC mice. (B) Crypts cultured in standard medium plus Wnt3a. Crypts from a control mouse showed rounded cysts. Exogenous Wnt3a did not support the growth of crypts from Zip7ΔIEC mice. Scale bar: 50 μm. (TIF) [file pgen.1006349.s005.tif]

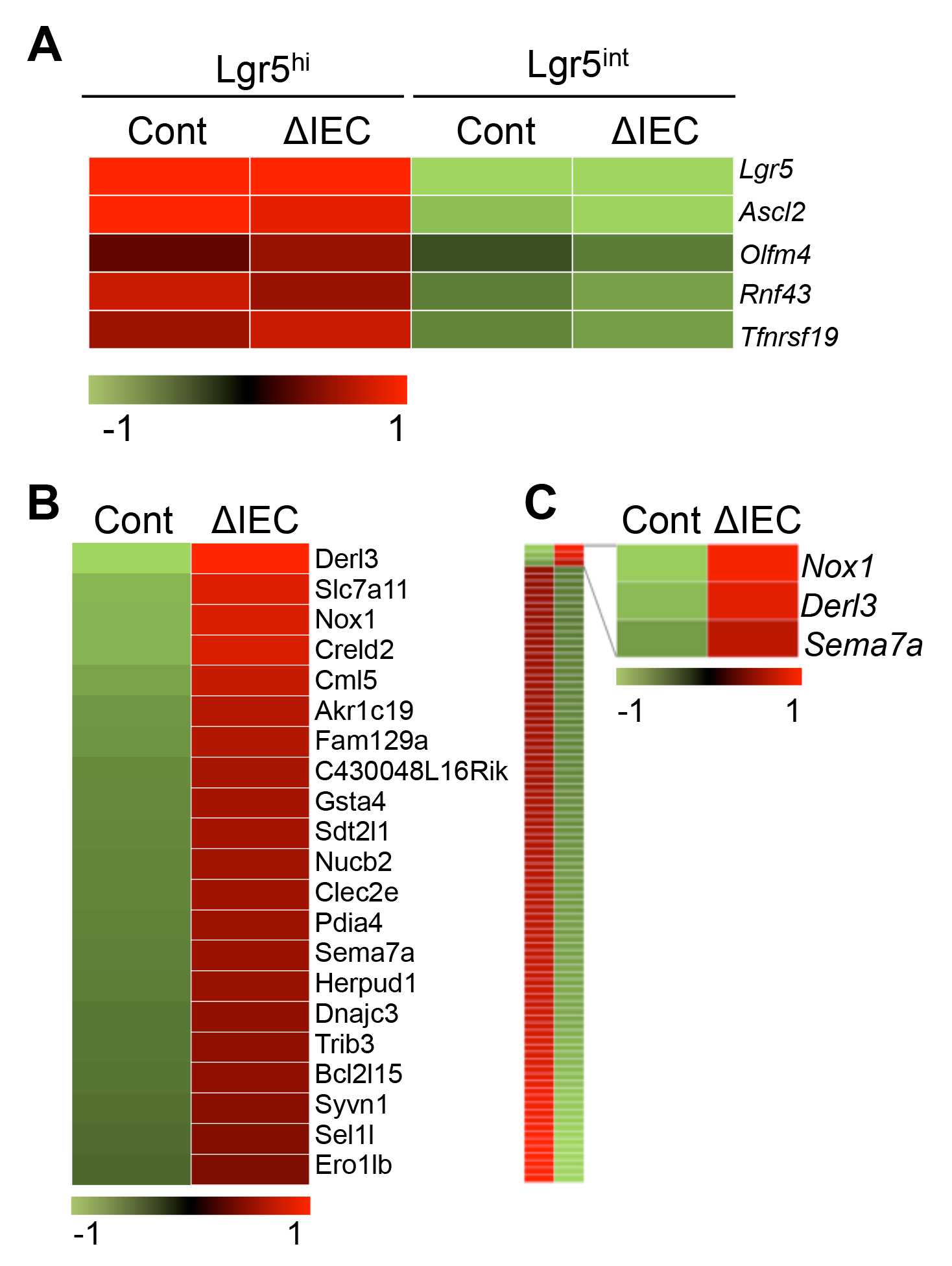

Supplement: S6 Fig — DNA microarray expression experiments were performed with sorted Lgr5hi and Lgr5int cells. (A) Heat map of stem cell-related genes in sorted Lgr5hi and Lgr5int cells from Zip7Cont and Zip7ΔIEC mice, showing a clear separation between the different Lgr5 populations. (B) Heat map of 1.8-fold change of gene epxression between Zip7Cont and Zip7ΔIEC Lgr5int cells. (C) Heat map showing differentially expressed transcripts in sorted Lgr5hi stem cells from Zip7Cont and Zip7ΔIEC mice 2 days after Cre activation. Columns represent different cell types, and rows represent differentially expressed genes. (TIF) [file pgen.1006349.s006.tif]

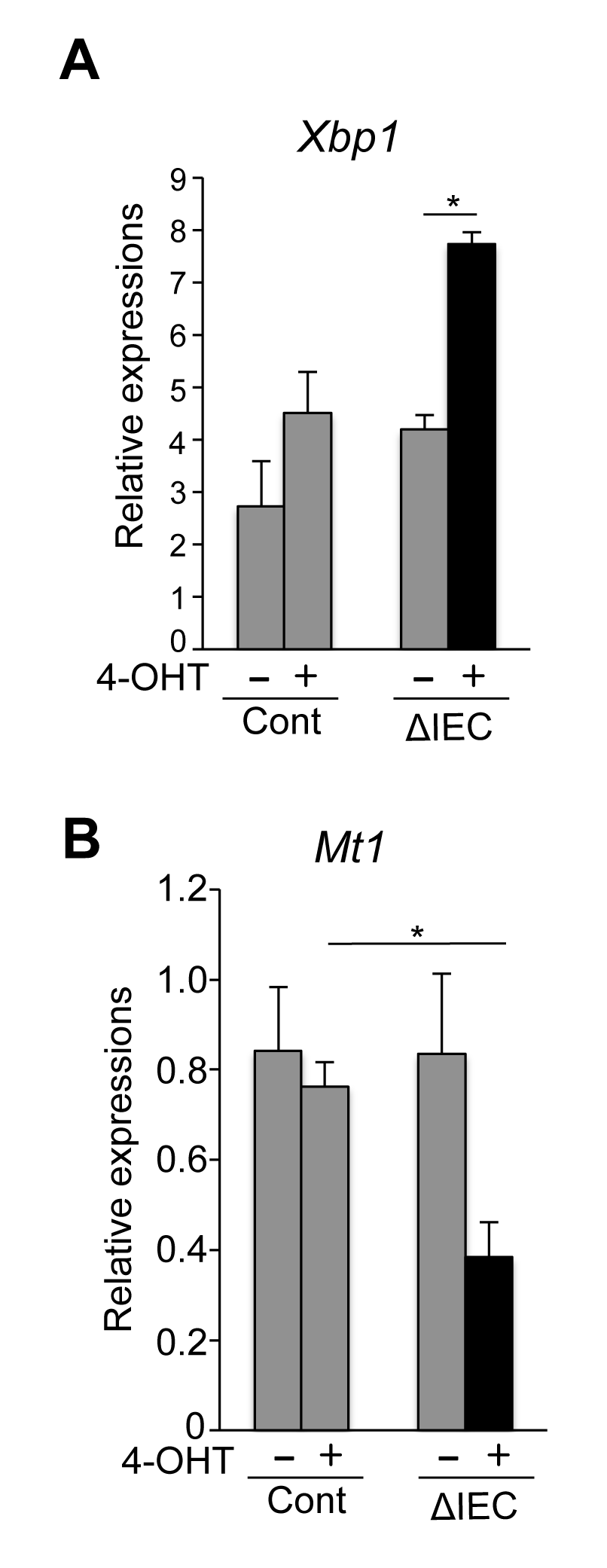

Supplement: S7 Fig — Quantitative PCR analysis of Xbp1 (A) and Mt1 (B) mRNA in cultured organoids from Zip7Cont and Zip7ΔIEC mice 48 h after 4-OHT treatment. (TIF) [file pgen.1006349.s007.tif]

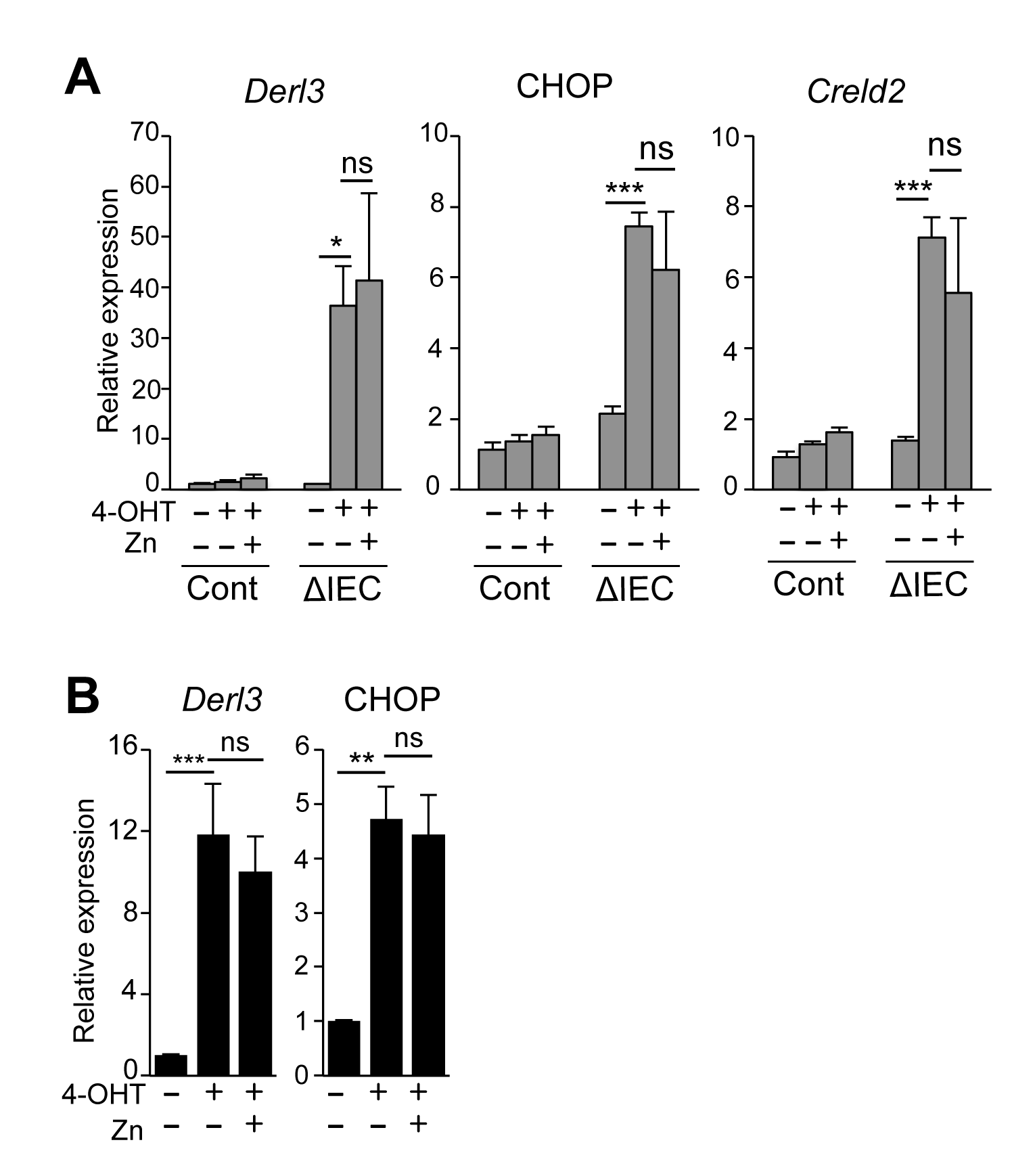

Supplement: S8 Fig — (A) Quantitative PCR analysis of Derl3, CHOP, and Creld2 in cultured organoids from Zip7Cont and Zip7ΔIEC mice 48 h after 4-OHT treatment with or without ZnSO4 (1 μM). (B) Quantitative PCR analysis of Derl3 and CHOP Zip7-/- MEF cells treated with 1μM of 4-OHT and ZnSO4 (1 μM). (TIF) [file pgen.1006349.s008.tif]

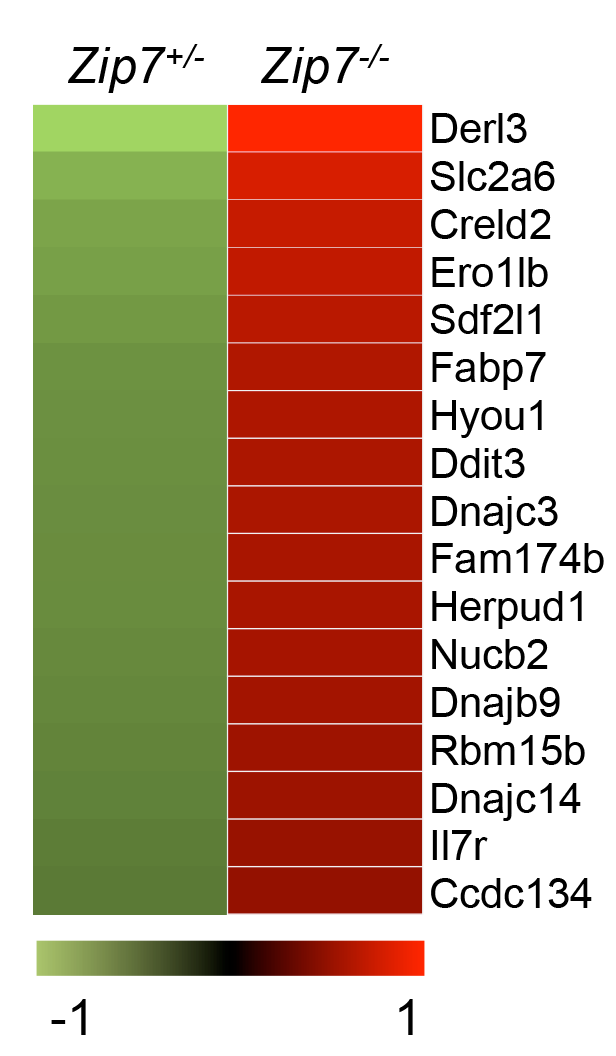

Supplement: S9 Fig — Heat map of 2.0-fold change of gene epxression between Zip7+/- and Zip7-/- MEF cells. Columns represent different cell types, and rows represent differentially expressed genes. (TIF) [file pgen.1006349.s009.tif]
