# Supplementary material for: Zinc Transporter SLC39A7/ZIP7 Promotes Intestinal Epithelial Self-Renewal by Resolving ER Stress
Source: PLoS Genet. 2016 Oct 13;12(10):e1006349. doi: 10.1371/journal.pgen.1006349 (PMC5065117; doi:10.1371/journal.pgen.1006349)
Supplement: S1 Table — (PDF) [file pgen.1006349.s010.pdf]

S1 Table Primer sequences of mouse genes used for quantitative real-time PCR.

Target gene	Primer	Sequence
SLC39A7/Zip7	Forward	5’-CTGGACATGGACACTCCCACA-3’
	Reverse	5’-AGGCGACAATCCCACTGAGAAC-3’
Krt20	Forward	5’-TTCAGTCGTCAAAGTTTTCACCG-3’
	Reverse	5’-TCCTATACAGCGAGCCACTCA-3’
Lgr5	Forward	5’-CTTCACTCGGTGCAGTGCT-3’
	Reverse	5’-CAGCCAGCTACCAAATAGGTG-3’
Ang4	Forward	5’-GCTGGGTCTGGTTGTGATTCC-3’
	Reverse	5’-AGGCGAGGTTAGCTTTCTTTCC-3’
Derl3	Forward	5’-ATGCTGGTCTATGTATGGAGCC-3’
	Reverse	5’-GTAAGCCGAAGAAGTTGACCC-3’
Derl1	Forward	5’-CATCACGCGCTACTGGTTTG-3’
	Reverse	5’-CCCACGGGGAAGTAAAAGGTG-3’
Slc2a6	Forward	5’-AACCGAGGGACTCGACTATGA-3’
	Reverse	5’-CAAGGCATACCCAAAGCTGAA-3’
Creld2	Forward	5’-CAACACGGCCAGGAAGAATTT-3’
	Reverse	5’-CATGATCTCCAGAAGCCGGAT-3’
*Ddit3(*CHOP*)*	Forward	5’-GTCCCTAGCTTGGCTGACAGA-3’
	Reverse	5’-TGGAGAGCGAGGGCTTTG-3’
Herpud1	Forward	5’-GCAGCCGGACAACTCTAATCA-3’
	Reverse	5’-AGAACTTCTCTTTGCCGTAAACC-3’
Mt1	Forward	5’-GCCTGCAAGAACTGCAAGTG-3’
	Reverse	5’-ATAGGAAGACGCTGGGTTGG-3’
